# A comprehensive analysis of Glasgow Prognostic Score (GPS)/the modified Glasgow Prognostic Score (mGPS) on immune checkpoint inhibitor efficacy among patients with advanced cancer

**DOI:** 10.1002/cam4.4940

**Published:** 2022-06-15

**Authors:** Yongchao Zhang, Shanshan Chen, Hualei Chen, Wei Li

**Affiliations:** ^1^ Cancer Center, Beijing Ditan Hospital Capital Medical University Beijing People's Republic of China

**Keywords:** cancer, glasgow prognostic score, immune checkpoint inhibitors, meta‐analysis, modified Glasgow prognostic score

## Abstract

**Background:**

The association between Glasgow Prognostic Score (GPS) and the modified Glasgow Prognostic Score (mGPS) and clinical outcomes in patients receiving immune checkpoint inhibitors (ICIs) remains controversial. Thus, this meta‐analysis aimed to examine the prognostic performance of GPS and mGPS in patients treated with ICIs.

**Methods:**

Eligible studies were retrieved from searches of EMBASE, PubMed, Web of Science, and Cochrane Library until July 2021. The hazard ratio (HR) and 95% confidence intervals (CIs) were pooled by using fixed‐effect or random‐effects model to evaluate the influence of GPS/mGPS on overall survival (OS) and progression‐free survival (PFS).

**Results:**

A total of 1164 patients were included. Overall, mGPS score of 2 and 1 were related to inferior OS (*p* < 0.001) and PFS (*p* < 0.001). Subgroup analyses showed no significant association between mGPS score of 1 and OS in patients with non‐small cell lung cancer (NSCLC), while this score was significantly associated with poor PFS in patients with NSCLC and head and neck squamous cell carcinoma. Higher GPS (score of 1 or 2) were associated with poor clinical outcomes (OS: *p* < 0.001; PFS: *p* = 0.036). Subgroup analysis showed high GPS levels were linked to worse OS in patients with NSCLC and gastric cancer, but not for PFS in these patients. Regarding test time point, GPS was related to worse OS and PFS in pre‐treatment GPS group, but not in post‐treatment GPS group.

**Conclusion:**

GPS and mGPS showed great potential to predict survival in patients treated with ICIs. Large and perspective trial are warranted to further validate these findings.

## Graphical abstract

In the present meta‐analysis, we pooled the data of 963 individuals from 11 studies on mGPS, and 505 patients from 4 GPS articles to assess the prognostic roles of mGPS and GPS among various cancer patients treated with ICIs. We found that higher mGPS and GPS were significantly related to inferior OS and PFS, indicating that these biomarkers may be as a clinical tool dichotomizing patients into responders and non‐responders to ICIs, and then assist clinicians to make decision regarding ordering of therapies.

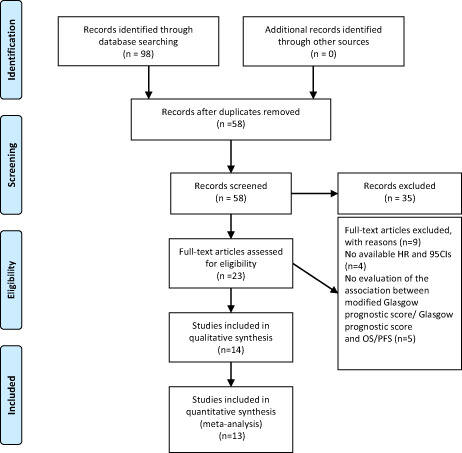

## 1 INTRODUCTION

Immune checkpoint inhibitors (ICIs) have reshaped the therapeutic management of various tumors, given durable tumor responses and manageable adverse effects.^1^ Nevertheless, the majority of patients less respond to ICIs with relatively low response rates to ICIs.^2^, ^3^ Thus, a biomarker capable of predicting clinical efficacy is critical for patient selection before immunotherapy.^4^

Increasing evidence supports that systematic inflammation responses are closely related to tumorigenesis, disease progression, and survival.^5^, ^6^ A variety of inflammatory biomarkers have ignited interest toward potential prognostic indices including neutrophil to lymphocyte ratio, c reactive protein (CRP), C‐reactive protein (CRP)/albumin ratio (CAR), Glasgow Prognostic Score (GPS), and the modified Glasgow Prognostic Score (mGPS).^7^, ^8^, ^9^, ^10^ CRP, an acute‐phase protein, increases quickly in response to systemic inflammation, and relates to worse survival in various cancers.^9^ Moreover, systemic inflammation and malnutrition can be captured by serum albumin, and hypoalbuminemia is a renowned predictor of poorer prognoses in cancer.^11^ Therefore, Forrest et al. initially incorporated both CRP and albumin, and developed the GPS for prognostication in patients with non‐small cell lung cancer (NSCLC).^12^ The GPS can be simply calculated according to a CRP level more than 1.0 mg/dl and serum albumin less than 3.5 g/dl, and each allocated a score of 1. Subsequently, since the inflammatory component of the score was found to be better associated with clinical benefits in various tumor types, the GPS was modified and was termed as mGPS. The disparity between mGPS and GPS is that hypoalbuminemia without increased CRP is not given a point.^13^

Recently, several studies have reported that high GPS/mGPS was associated with worse survival outcomes in patients with various cancers, regardless of surgery or chemotherapy.^14^, ^15^ However, in the era of immunotherapy, it remains to be determined whether such immune‐related indices reflect the response to ICIs due to some contradictory results. We thus performed this meta‐analysis to investigate the correlation between baseline GPS/mGPS and survival of cancer patients receiving ICIs.

## 2 MATERIALS AND METHODS

### 2.1 Search strategies

We conducted a comprehensive electronic search employing PubMed, Web of science, EMBASE, and Cochrane Library up to June 23, 2021. The keywords were as follows: “immune check point inhibitor*”, “neoplasm”, “Glasgow prognostic score or modified Glasgow prognostic score” or “GPS” and “mGPS”, “CTLA‐4”, “ipilimumab”, “PD‐1”, “PD‐L1”, “nivolumab”, “avelumab,” “durvalumab”, “atezolizumab”, “pembrolizumab”, “immune checkpoint inhibitor”, “immunotherapy”, “prognosis”, “prognostic”, and “survival”.

### 2.2 Study selection criteria

Studies that met the following requirements were included: (1) studies including patients with tumors receiving ICIs; (2) reporting data regarding the hazard ratio (HR) and 95% confidence interval (CI) for overall survival (OS) or progression‐free survival (PFS). The following exclusion criteria were applied: (1) Letters, conference abstracts, editorials, case reports, and reviews; (2) insufficient information to calculate HRs and 95% Cis; (3) Studies were not in English.

### 2.3 Data extraction and quality assessment

The following information was extracted independently by two reviewers: first author, publication year, country, study design, sample size, treatment, the score of GPS or mGPS, HRs for OS and PFS, and 95% CIs. We assess the quality of included studies with the Quality Assessment of Newcastle‐Ottawa Scale (NOS), which includes three aspects: selection, comparability, and outcome assessment. Included studies assigned with scores higher than 5 were high‐quality studies. Disparities were addressed by discussion.

### 2.4 Statistical analysis

Pooled HR were calculated based on HRs with their 95% CIs. Heterogeneity of the included studies was evaluated by Higgins *I* ^2^ statistic. *I* ^2^ > 50% was represented significant heterogeneity, and then a random effect model was used. Otherwise, a fixed‐effect model was applied. The sources of heterogeneity were examined by sensitivity and subgroup analysis. Funnel plots and Egger's test were utilized to identify publication bias. Statistical significance was defined as *p* < 0.05 for all statistical tests with STATA 16.0 (Stata Corp.).

## 3 RESULTS

### 3.1 Study selection and characteristics

**FIGURE 1:**
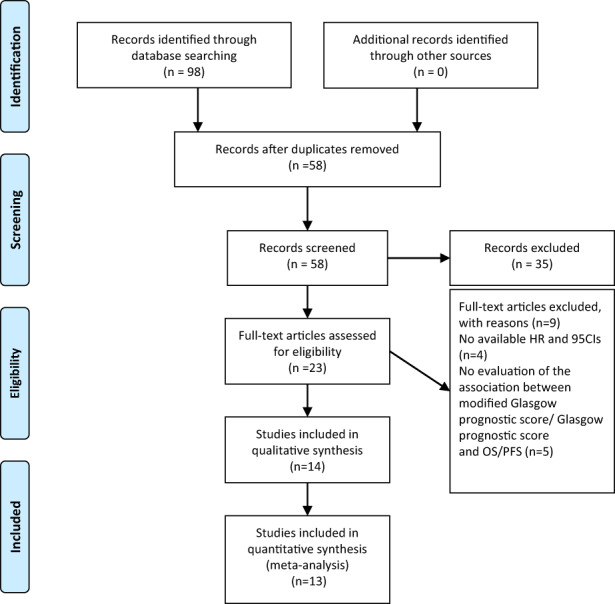
Flow chart of literature search and study selection. A total of 98 hits were initially retrieved. After carefully screened 14 studies reporting the impact of mGPS/GPS on the survival of patients with cancer treated with ICI were included in the analysis.

A total of 98 relevant hits were initially obtained from selected databases. There were 58 records remained after removing duplicates. Of these, 35 articles were discarded by titles and abstracts review due to reviews, conference abstracts, irrelevant studies, letters, or case reports. The full texts of the remaining 23 articles were reviewed. Finally, 14^16^, ^17^, ^18^, ^19^, ^20^, ^21^, ^22^, ^23^, ^24^, ^25^, ^26^, ^27^, ^28^, ^29^ studies including 1164 patients were eligible (Figure 1). Among them, most studies considered mGPS as categorical variable in multivariate analysis, while Takamori et al.^27^ used mGPS as a continuous variable, and therefore this study was not enrolled in meta‐analysis. The main characteristics of studies included in this meta‐analysis were shown in Table 1.

**TABLE 1:** The main characteristics of studies included in this meta‐analysis

ID	Country	Cancer type	ICIs treatment	Sample size	Line of treatment	GPS/mGPS	Score (*n*, %)	Outcome	Analysis model	Time point	NOS
Araki 2021	Japan	NSCLC	Nivolumab	116	Second	mGPS	0 39 34.5% 1 37 32.7% 2 37 32.7%	OS	Univariate	Pre	6
Brown 2021	US	Metastatic urothelial carcinoma	Atezolizumab Pembrolizumab Nivolumab	53	First Second Third	mGPS	0 23 43.4% 1 15 28.3% 2 15 28.3%	PFS/OS	Multivariate	Pre	6
Brown 2021	US	Metastatic renal cell carcinoma	Anti‐ PD‐1 Anti‐ PD‐1 + anti‐ CTLA‐4 Anti‐PD‐1/PD‐L1 + anti‐VEGF Anti‐ PD‐1 + experimental therapy	156	First‐line second‐line further‐line	mGPS	0 57 36.6% 1 62 39.7% 2 37 23.7%	PFS/OS	Multivariate	Pre	
Freitas 2021	Portugal	NSCLC	Nivolumab Pembrolizumab	77	1 ≥2	mGPS	0 20 26.0% 1 14 18.2% 2 11 14.3%	PFS/OS	Multivariate	Pre	6
Fujiwara 2020	Japan	Metastatic renal cell carcinoma	Nivolumab	45	First Second Third	mGPS	0 29 64% 1 8 18% 2 8 18%	PFS/OS	Multivariate	Pre	7
Kasahara 2019	Japan	NSCLC	Nivolumab Pembrolizumab	47	1–2/≥3	GPS post	0 24 51.0% 1 6 12.8% 2 17 36.2%	PFS/OS	Pre‐univariate/post‐multivariate	Pre/Post	6
Kurosaki 2020	Japan	Gastric Cancer	Nivolumab	80	A third‐ or laterline	GPS	0 29 64% 1 8 18% 2 8 18%	PFS/OS	Multivariate	Pre	6
Matsubara 2020	Japan	NSCLC	Atezolizumab	24	Second‐ or further‐line	mGPS	0–1 13 54.2% 2 11 45.8%	OS	Multivariate	Pre	5
Matsuki 2021	Japan	Head and neck squamous cell carcinoma	Nivolumab	88	Second	mGPS	0 41 47% 1 25 28% 2 22 25%	PFS/OS	Multivariate	Pre	6
Namikawa 2020	Japan	Gastric cancer	Nivolumab	29	Second‐ or further‐line	GPS	0 29 64% 1 8 18% 2 8 18%	PFS/OS	Multivariate	Pre/Post	5
Niwa 2020	Japan	Salivary gland carcinoma	Nivolumab	24	Second‐ or further‐line	mGPS	0–1 14 58.3% 2 10 41.7%	PFS/OS	Univariate	Pre	5
Ogura 2021	Japan	NSCLC	Nivolumab	34	First Second	mGPS	NA	PFS/OS	Univariate	Pre/Post	6
Takamori 2021	Japan	NSCLC	Nivolumab Pembrolizumab Atezolizumab	304	First or second Third or higher	GPS/mGPS	0109 35.9% 1 85 28.0% 2110 36.1%	PFS/OS	Univariate/Multivariate	Pre	5
Tokuyama 2021	Japan	Gastric cancer	Nivolumab	45	Second‐ or further‐line	GPS	0 25 55.5% 1 18 40% 2 2 4.5%	OS	Univariate	Pre	6
Ueki 2021	Japan	Head and neck squamous cell carcinoma	Nivolumab	42	Second‐ or further‐line	mGPS	0 24 57.1% 1 7 16.7% 2 11 26.2%	OS	Multivariate	Pre	

Abbreviations: OS, overall survival; PFS, progression‐free survival; NSCLC, non‐small cell lung cancer;; RCC, renal cell carcinoma.

Six studies focused on patients with NSCLC, three studies on gastric cancer, two articles on head and neck squamous cell carcinoma, two studies on renal cell carcinoma, and two studies on urothelial carcinoma and salivary gland carcinoma, respectively. Most studies are conducted in Japan, and all included studies are retrospective. The types of ICIs involved in our studies only comprised PD‐1/PD‐L1 inhibitors (Nivolumab, Pembrolizumab, and Atezolizumab) except for one study, which included ICI as monotherapy or in combination with anti‐ CTLA‐4 and anti‐VEGF treatments.

Regarding testing time of GPS/mGPS, most studies assessed pre‐treatment mGPS, and only one study evaluated post‐treatment mGPS. Similarly, only two studies evaluated post‐treatment GPS. Besides, the majority of patients in our study WERE treated with ICIs as first‐or further‐line settings.

### 3.2 Prognostic impact of mGPS on survival outcomes

**FIGURE 2:**
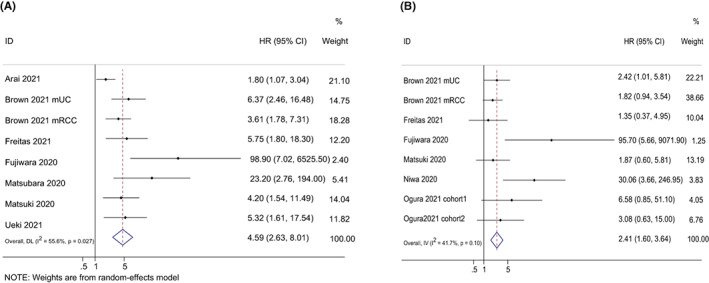
Forest plots of the hazard ratios and 95% CIs for overall survival of mGPS score of 2 (A) and mGPS score of 1 (B) in patients treated with ICI. Abbreviation: mGPS, modified Glasgow prognostic score; HR, hazard ratio; CI, confidence interval.

According to test time point (pretreatment or posttreatment), we treated Ogura 2021^26^ as two separate studies—‘Ogura2021cohort 1’ and ‘Ogura2021cohort 2. Overall, mGPS score of 2 from eight studies were associated with poor OS in patients receiving ICIs (HR = 4.59, 95% CI: 2.63–8.01, *p* < 0.0001), with significant heterogeneity (*I* ^2^ = 55.6%). Moreover, mGPS score of 1 from seven studies were found to be correlated to worse OS (HR = 2.41, 95% CI: 1.60–3.64, *p* < 0.001), with moderate heterogeneity (*I* ^2^ = 41.7%) (Figure 2). Subgroup analyses were conducted upon cancer type. Our pooled results showed that mGPS score of 2 was consistently related to inferior OS among various cancers. Notably, mGPS score of 1 was no significant association in patients with NSCLC (shown in Table 2).

**TABLE 2:** Results of subgroup analysis

Analysis	*N*	OS			*N*	PFS		
		Association		Heterogeneity		Association		Heterogeneity
		HR (95% CI)	*p*	*I* ^2^		HR (95% CI)	*p*	*I* ^2^
mGPS score of 2								
Total	8^a^	4.59 (2.63–8.01)	0.001	55.6%	4^a^	2.42 (1.72–3.42)	0.001	41.0%
Cancer type								
NSCLC	3	4.61 (1.25–16.99)	0.022	74.1%	–	–	–	–
mRCC	2	4.13 (2.07–8.26)	0.001	71.1%	–	–	–	–
Head and neck squamous cell	2	4.63 (2.15–9.99)	0.001	0%	–	–	–	–
mGPS score of 1								
Total	7^b^	2.41 (1.60–3.64)	0.001	41.7%	5	1.74 (1.27–2.40)	0.001	25.2%
Cancer type								
NSCLC	3	2.40 (0.97–5.91)	0.058	0%	3	2.61 (1.28–5.34)	0.008	0%
mRCC	2	2.06 (1.07–3.96)	0.03	76.7%	**–**	**–**	**–**	**–**
GPS								
Total	6	3.89 (1.47–7.99)	0.001	66.8%	5	1.48 (1.03–2.13)	0.036	42.8%
Cancer type								
NSCLC	2	3.42 (1.47–7.99)	0.004	37.1%	2	1.63 (0.95–2.78)	0.74	14.3%
Gastric cancer	4	4.36 (1.26–15.00)	0.02	77.7%	3	1.36 (0.82–2.24)	0.233	64.2%
Time point								
Pre	4	5.11 (2.30–11.38)	0.001	48.4%	3	1.67 (1.04–2.67)	0.033	0%
Post	2	2.30 (0.43–12.43)	0.332	83.4%	2	1.23 (0.69–2.19)	0.486	80.3%

Abbreviations: OS, overall survival; PFS, progression‐free survival; NSCLC, non‐small cell lung cancer; RCC, renal cell carcinoma.

**FIGURE 3:**
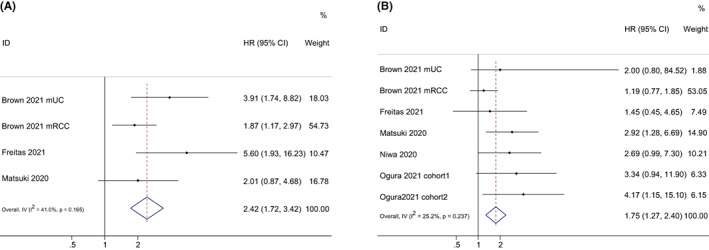
Forest plots of the hazard ratios and 95% CIs for progression‐free survival of mGPS score of 2 (A) and mGPS score of 1 (B) in patients treated with ICI. CI, confidence interval; HR, hazard ratio; mGPS, modified Glasgow prognostic score.

Evaluation of the correlation between mGPS score of 2 and 1 and PFS were reported in four studies and six studies, respectively. Overall, pooled results revealed that mGPS score of 2 (HR = 2.42, 95% CI = 1.72–3.42, *p* < 0.001) and of 1 (HR = 1.74, 95% CI = 1.27–2.40, *p* < 0.001) were related to inferior PFS, with moderate heterogeneity (score of 2: *I* ^2^ = 41.0%; score of 1: *I* ^2^ = 25.2%) (Figure 3). Then, subgroup analyses were performed according to cancer types. The subgroup analysis of mGPS score of 2 stratified by cancer types was not performed, with four studies reporting HRs from patients with four cancer types, while mGPS of 1 were found to be significantly related to poor PFS in patients with NSCLC and head and neck squamous cell carcinoma (shown in Table 2).

Sensitivity analyses of mGPS for the OS and PFS were conducted to determine whether an individual study had an effect on the results. There was no significant influence on results, regardless of mGPS score of 2 or 1, suggesting that these results were robust. The funnel plots involving mGPS for OS indicated that obvious publication bias existed, regardless of mGPS score of 2 or 1 (see in Figures S1 and S2). The results were confirmed by Egger's test (score of 2: *p* < 0.001, score of 1: *p* = 0.018). For PFS, the funnel plots of mGPS score of 2 or 1 were symmetrical, indicating no potential publication bias (see Figures S3 and S4), which was further validated by Egger's test (*p* = 0.185 and *p* = 0.073 for score of 2 and 1, respectively).

### 3.3 Prognostic impact of GPS on survival outcomes

**FIGURE 4:**
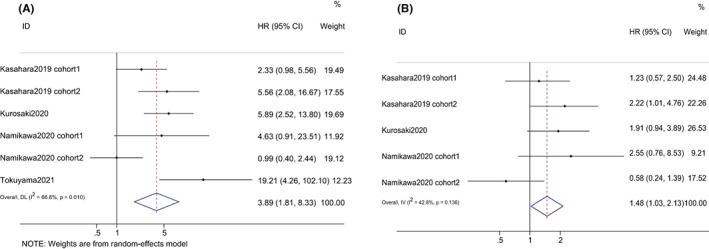
Forest plots of the hazard ratios and 95% CIs for overall survival (A) and progression‐free survival (B) of GPS in patients treated with ICI. GPS, Glasgow prognostic score; HR, hazard ratio; CI, confidence interval.

Both Kasahara and Namikawa investigated the effect of GPS on OS and PFS before and after ICIs, therefore we considered Kasahara2019^20^ and Namikawa2020^24^ as four separate studies—Kasahara2019 cohort 1, Kasahara2019 cohort 2, Namikawa2020 cohort 1 and Namikawa2020 cohort 2. The prognostic performance of GPS on OS was evaluated in four studies. Overall, higher GPS (score of 1 or 2) were associated with poor OS (HR = 3.89, 95% CI: 1.81–8.33, *p* < 0.001), with significant heterogeneity (*I* ^2^ = 66.8%) (Figure 4). Unlike mGPS, meta‐analysis based on score of 2 or 1 were not performed due to limited studies. In subgroup analysis stratified by cancer types, high GPS was related to worse OS in patients with NSCLC and gastric cancer. In terms of test time point, a significant correlation between GPS and OS in pre‐treatment GPS group was obtained, but not in post‐treatment GPS group, with pooled HRs of 5.11 (95% CI: 2.30–11.38, *p* = 0.001), and 2.30 (95% CI: 0.43–12.43, *p* = 0.332).

The relationship between GPS and PFS was revealed in three studies. Similarly, higher GPS were also linked to worse PFS (HR = 1.48, 95% CI: 1.03–2.13, *p* = 0.036), with moderate heterogeneity (*I* ^2^ = 25.2%). Subgroup analysis on the basis of cancer types revealed that there was no statistically significant association between high GPS levels and inferior PFS in patients with NSCLC and gastric cancer, with pooled HRs of 1.63 (95% CI: 0.95–2.78, *p* = 0.074), and 1.36 (95% CI: 0.82–2.24, *p* = 0.233). With regard to test time point, like OS, high GPS was related to worse PFS in pre‐treatment GPS group, but not for post‐treatment GPS group with pooled HRs of 1.67 (95% CI: 1.04–2.67, *p* = 0.033), and 1.23 (95% CI: 0.69–2.19, *p* = 0.486) (shown in Table 2).

Sensitivity analyses revealed that no individual study could substantially affect the combined HRs and its 95% Cis for the OS. Nevertheless, for the PFS, pooled results were significantly influenced when Kasahara cohort 2^20^ and Namikawa cohort 1^24^ were excluded. The funnel plot of GPS for OS and PFS indicated no publication bias (see Figures S5 and S6). The results were further confirmed by Egger's test (OS: *p* = 0.303; PFS: *p* = 0.950).

## 4 DISCUSSION

In the present meta‐analysis, we pooled the data of 963 individuals from 11 studies on mGPS, and 505 patients from four GPS articles to assess the prognostic roles of mGPS and GPS among various cancer patients treated with ICIs. We found that higher mGPS and GPS were significantly related to inferior OS and PFS, indicating that these biomarkers may be as a clinical tool for dichotomizing patients into responders and non‐responders to ICIs, and then assisting clinicians to make decision regarding the ordering of therapies. To our best knowledge, this is the first comprehensive analysis to evaluate the association between mGPS/GPS and survival outcomes in patients treated with ICIs.

The advent of ICIs has reshaped the management of many tumor types, especially for those cancers by a lack of treatment options. Nevertheless, a great number of tumor patients fail to respond to ICIs, creating an urgent need to identify prognostic biomarkers capable of predicting prognosis in these patients.^1^ Although some biomarkers such as PD‐L1 levels in tumor tissue, tumor mutation burden, and CD8^+^ tumor‐infiltrating lymphocytes, have been reported to be predictive of the efficacy of ICIs, these markers have yet to be established due to no conclusive results across tumors.^30^, ^31^, ^32^ In addition, these biomarkers are inconvenient for tumor tissue are not easy to available and hard to assess as a time series. Accordingly, an easy‐to‐use biomarker capable of noninvasive evaluation is needed.

The GPS/mGPS, which comprise CRP and albumin, are composite biomarkers of systemic inflammation and nutritional status. The critical role of systemic inflammation in the development and progression of tumor has been widely accepted. A great number of studies reported that hematological parameters which can capture inflammatory conditions, such as neutrophils, lymphocytes, platelets, and CRP, are related to prognosis in cancer patients.^9^ Elevated serum CRP is triggered by IL‐6 that has been demonstrated to promote resistance to ICIs. Moreover, high CRP has been demonstrated to be related to low levels of CD4^+^ T‐cells, which is of importance in antitumor immune response to ICIs.^33^ Undernutrition captured by albumin is another determinant of poor prognosis in cancer patients, and malnutrition can reduce tolerance to adverse events following ICIs. Moreover, albumin level is also an indicator of inflammation. Low albumin levels are often induced by chronic inflammation due to the increased vascular permeability of albumin and decreased hepatic albumin production. Prostaglandin E2 (PGE2), which weakens the ability and decreases immune cells, can be activated by albumin, resulting in immune suppression microenvironment.^34^ Taken together, a higher GPS/mGPS, which reflects elevated CRP and/or decreased albumin, is a promising biomarker to predict response to ICIs.

Although the prognostic roles of mGPS/GPS in patients treated with ICIs have started to be recognized and investigated in recent years, there are open questions about the standardized use of mGPS/GPS. First, mGPS/GPS should be hierarchic as these is characterized by ordinal categorical variables. GPS/mGPS of 0, 1, and 2 should be considered as low, moderate and high risk. In our study, we pooled HRs based on mGPS equal to 1 or 2, referring to mGPS of 0 for differentiating between the different levels. Regarding GPS, although we cannot conduct this like mGPS due to limited studies, we still demonstrated that higher GPS was associated with poor prognosis. Second, the treatment history may affect the mGPS/GPS. Since CRP and albumin are dynamic biomarkers that can be affected by many factors, including underlying diseases, treatment approaches (resection or radiotherapy), and testing conditions.^15^, ^35^ Third, CRP and albumin might alter over time points during the treatment of ICIs. Therefore, apart from treatment history, a post‐treatment analysis can also capture the changes in immune response. For patients with NSCLC, mGPS score of 1 was not associated with OS, but associated with worse PFS. Nevertheless, higher GPS was linked to poor OS, but not for PFS in patients with NSCLC. This may be partly explained by, in our study, only Ogura et al.^26^ reported post‐treatment mGPS was associated with OS and only Kasahara et al.^20^ indicated that GPS was associated with survival outcomes. And different test point time (pretreatment or posttreatment) may affect albumin and CRP. Forth, cancer type may influence immune microenvironment. For GPS, Kasahara^20^ reported post‐treatment GPS was significantly related to worse survival in patients with NSCLC, while Namikawa^24^ showed no significant association in patients with gastric cancer. Thus, well‐designed clinical trials are needed to further investigate the concerns above.

Several limitations should be acknowledged. First, all of the included studies were retrospective, whose results might be influenced by selection bias and reporting bias. The GPS/mGPS need to be demonstrated in perspective studies. Second, the majority of the recruited patients from eligible studies are Japanese, while only three studies were performed in Western countries. Third, although subgroup analysis based on types of cancers has been conducted, relevant studies were insufficient in specific cancer types, which need to cautiously generalize these results in these patients. Forth, comprehensive subgroup analyses according to other confounders were not done due to the small sample size, which may bias our results. Fifth, the line of ICIs varies according to different tumor types. Therefore, previous treatments may have affected CRP and albumin levels at the initiation of immunotherapy.

## 5 CONCLUSION

Our study showed that higher mGPS/GPS were associated with poor prognosis in patients undergoing ICIs. Types of cancer, testing time may affect the prognostic performance of mGPS/GPS. These results demonstrated that the mGPS/GPS as a convenient and cost‐effective score derived from blood measured routinely may help clinicians to properly use ICIs to treat patients with cancer.

## AUTHOR CONTRIBUTIONS

Yongchao Zhang analyzed the data and wrote the first draft. Shanshan Chen and Hualei Chen analyzed the data. WeiLi designed the study, proof read and revised the submission. All authors discussed the results and approved the final manuscript.

## FUNDING STATEMENT

This study is funded by Beijing Talents Project and Capital's Funds for Health Improvement and Research (grant number 2020–2475 2175).

## CONFLICT OF INTEREST

The authors declare that they have no known competing financial interests or personal relationships that could have appeared to influence the work reported in this paper.

## Supporting information


Figure S1
Figure S2
Figure S3
Figure S4
Figure S5
Figure S6

## Data Availability

Upon request.
