# Supplementary material for: A comprehensive analysis of Glasgow Prognostic Score (GPS)/the modified Glasgow Prognostic Score (mGPS) on immune checkpoint inhibitor efficacy among patients with advanced cancer
Source: Cancer Med. 2022 Jun 15;12(1):38–48. doi: 10.1002/cam4.4940 (PMC9844653; doi:10.1002/cam4.4940)
Supplement: Supplementary file 1 — Figure S1 Figure S2 Figure S3 Figure S4 Figure S5 Figure S6 [file CAM4-12-38-s001.docx]

**Supplement**


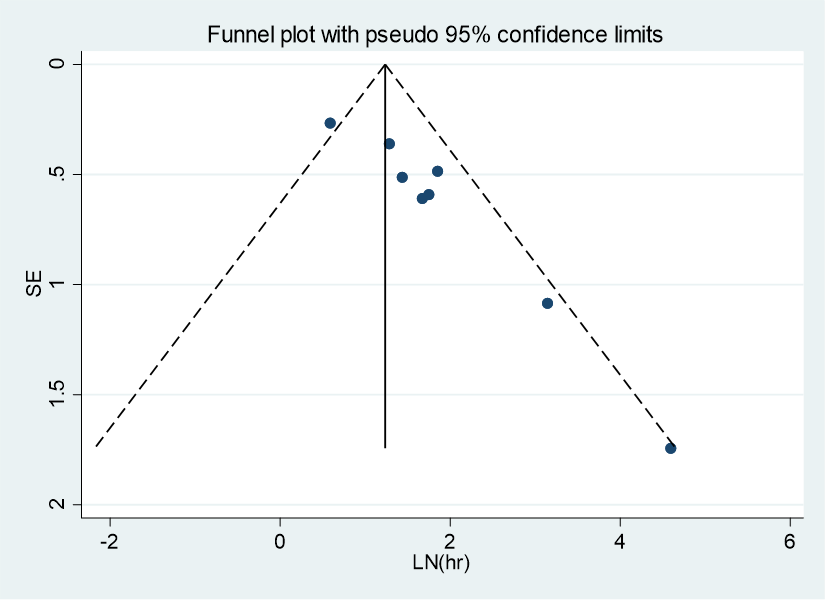
**Figure 1** ***Funnel plots of the association between mGPS 2 and OS***

**Figure 2** ***Funnel plots of the association between mGPS 1 and OS***

**Figure 3** ***Funnel plots of the association between mGPS 2 and PFS***

**Figure 4 *Funnel plots of the association between mGPS 1 and PFS***

**Figure 5 *Funnel plots of the association between GPS and OS***

**Figure 6 Funnel plots of the association between GPS and PFS**
